# Birth-related and current factors associated with physical inactivity in the leisure time in Brazilian adolescents

**DOI:** 10.1371/journal.pone.0273611

**Published:** 2022-09-09

**Authors:** Maria Laura Siqueira de Souza Andrade, Juliana de Souza Oliveira, Nathália Paula de Souza, Emilia Chagas Costa, Fernanda Cristina de Lima Pinto Tavares, Poliana Coelho Cabral, Nathalia Barbosa de Aquino, Vanessa Sá Leal, Pedro Israel Cabral de Lira

**Affiliations:** 1 Department of Nutrition, Universidade Federal de Pernambuco, Recife, Pernambuco, Brazil; 2 Nutrition Center, Centro Acadêmico de Vitória, Universidade Federal de Pernambuco, Vitória de Santo Antão, Pernambuco, Brazil; Yamaguchi University: Yamaguchi Daigaku, JAPAN

## Abstract

**Background:**

To determine whether biological and sociodemographic factors at birth and current factors are associated with insufficient physical activity during leisure among Brazilian adolescents.

**Methods:**

A school-based cross-sectional study with national coverage was conducted involving Brazilian adolescents 12 to 17 years of age in municipalities with more than 100 thousand residents. The sample consisted of 74,589 adolescents who participated in the Study of Cardiovascular Risk in Adolescents. Insufficient leisure-time physical activity was categorized based on total volume (<300 minutes/week = insufficiently active; >300 minutes/week = sufficiently active). Poisson regression models were used to assess associated factors.

**Results:**

Most adolescents were classified as insufficiently active (54.8%; 95%CI: 53.7–55.9). The variables associated with insufficient physical activity during leisure were the female sex (70.4%; 95%CI: 68.8–71.9), age between 15 and 17 years (57.8%; 95%CI: 56.3–59.2), pertaining to the low or middle class (54.5%; 95%CI: 52.8–56.1), and not being overweight (55.9%; 95%CI: 54.6–57.1).

**Conclusion:**

Contrary to our hypothesis, birth related factors (e.g., low birth weight, preterm birth and exclusive breastfeeding until 6 months of age) are not associated with physical inactivity. The prevalence of insufficient physical activity during leisure was high among the adolescents evaluated and was associated with sociodemographic characteristics as well as nutritional status. It is necessary to implement strategies focused on physical activity at schools.

## Introduction

Adolescence is a critical period for the development of unhealthy habits, such as physical inactivity, which is considered a pandemic in the field of public health [1]. Physical inactivity in this phase may be influenced by biological factors at the onset of life, such as low birth weight [2] and premature birth [3].

The explanation for this influence is that the insufficient offer of nutrients in the intrauterine environment causes different morphological and physiological responses during fetal development [4]. Such responses seem to program behavior with regards to the practice of physical activity in adolescence [5], which, in turn, increases the risk of chronic non-communicable diseases in adulthood. [4].

Current aspects and/or determinants are also associated with the insufficient practice of physical activity in this age group, such as sociodemographic, biological, psychological, cultural, and environmental factors. [6, 7] Those most described in the literature are the female sex [8], a low socioeconomic status [6, 8], an older age [6, 8], and the high consumption of foods rich in sugar and fat [7].

Beside current factors, there is the influence of birth-related variables on the definition of aspects related to lifestyle and health throughout the phases of life [5]. A meta-analysis involving 43,442 adolescents and adults found that adolescents having been born with a low birth weight spent less time on physical activity in the leisure context [2]. However, few national and international studies have considered this approach through an analysis of different outcomes in the field of physical activity. Indeed, most studies have addressed different chronic non-communicable diseases or physiological health outcomes [9].

It is therefore important to determine the level of physical activity in adolescence according to current factors and those related to the onset of life. The findings of such an investigation would lead to a better understanding of the factors that mediate these relations as well as enable the proposal of public policies and more effective strategies based on the actual situation of the adolescent population [1, 10].

The hypotheses of this study were 1) Unfavorable biological, behavioral and sociodemographic factors in early life, such as low birth weight, preterm birth and exclusive breastfeeding until 6 months of age, are associated with physical inactivity in adolescents Brazilians. 2) Current sociodemographic factors (low socioeconomic status, lower maternal schooling, among others) will be associated with the highest percentage of adolescents classified as inactive.

Therefore, the aim of the present study was to determine whether biological and sociodemographic factors at birth and current factors are associated with the insufficient practice of physical activity during leisure among Brazilian adolescents.

## Methods

### Design and sample

A cross-sectional study was conducted with male and female adolescents 12 to 17 years of age enrolled at public and private schools in the morning and afternoon shifts in 273 Brazilian cities with more than 100 thousand residents. The adolescents were participants of the *Estudo de Riscos Cardiovasculares em Adolescentes* (ERICA [Study of Cardiovascular Risk in Adolescents]) [11, 12].

The participants were selected using a stratified sampling procedure in three stages. In each geographic stratum, schools were selected with probability proportional to size. A total of 32 strata were considered, involving 27 large cities and five sets of municipalities with more than 100 thousand residents in each of the five geographical macro-regions of the country. At the selected schools, a survey was made of classes and students in the 7^th^, 8^th^, and 9^th^ years of primary school as well as the 1^st^, 2^nd^, and 3^rd^ years of high school to select the equivalent of three classes at each school. All students in the selected classes were asked to participate in the study.

After the selection of the sample, data were obtained from 74,589 adolescents of 1247 schools in 124 Brazilian cities. For further information on the sampling design and procedures adopted in the ERICA study, see the studies conducted by Bloch et al. [11] and Vasconcellos et al. [12], respectively.

In the self-administered form by the students, the age at full years and sex. Information on date of birth and age was used to identify adolescents eligible for the study, since students with younger ages 12 years old and over 17 years old could be enrolled in the selected classes.

Were excluded for analysis teenagers who are just outside the 12 to 17 age group, such as pregnant teenagers and those with physical, size or permanent disabilities, which do not allow measurement of anthropometric measurements with the instruments used in the research.

### Data collection and measurement instrument

Data collection was carried out between February 2013 and November 2014 by a previously trained team composed of different healthcare providers. Two validated questionnaires were used [11, 12], which were self-administered by the parents or adolescents using personal digital assistants. The questionnaire addressed sociodemographic, behavioral, and health-related characteristics.

For data collection, two questionnaires were used, one for students and another for parents/ care giver’s as presented BLOCH et al. [11]. The students’ questionnaire was self-completed by the adolescents using electronic devices (personal digital assistants—PDA) and contained questions referring to health behaviors, as described below: 1) sociodemographic information on adolescents; 2) alcoholism and smoking; 3) eating habits; 4) work activity; 5) reproductive health; 6) physical activity; 7) hours of sleep.

The Parents’ questionnaire included questions about: 1) socioeconomic, family and housing information of the parents and/or care giver’s; 2) previous and current history of cardiovascular and metabolic diseases in the family; 3) Information on the adolescent’s birth (weight at birth, gestational age and breastfeeding of the adolescent) [11, 12]. The printed form was sent to the parents’/caregivers’ by the students. This is the only information source that was collected using a hard (printed) form. Data was double entered to avoid typing errors. Prior to the beginning of data collection, a pilot study was carried out in order to test the consistency of measures of the instrument (questionnaire) used and its applicability regarding the time of application, acceptance and understanding of the questions by the people interviewed. The pilot study was conducted with adolescents and their parents in schools in cities in different regions.

### Description of the independent variables of the study

#### Biological and behavioral factors in early life

The following biological and behavioral factors related to the onset of life were investigated: 1) Birth weight: low weight (< 2500g); insufficient weight (2500 to 2999g); adequate weight (3000 to 3999g), and high weight (≥ 4000g) [13]; 2) duration of pregnancy: <8 months or 9 to 10 months; 3) duration of exclusive breastfeeding: <3 months; 3 to 6 months; or >6 months; and 4) mother’s age during pregnancy: <25 years; 25 to 35 years; or >35 years.

#### Sociodemographic data

The following current characteristics were investigated: 1) Adolescent’s sex (male or female); 2) adolescent’s age (12–14 year or 15–17 years); 3) mother’s schooling (≤ 4 years; 5 to 8 years, or >8 years); 4) Family income *per capita*, sum of incomes of family member and categorized using on the criteria proposed by the *Associação Brasileira de Empresas de Pesquisa* (ABEP [Brazilian Association of Research Firms] [14], which classifies families as follows: High income (Classes A1 and A2), middle income (Classes B1, B2 and C1),and low income (Classes C2, D and E); 5) type of school (public or private); 6) geographic stratum (metropolis or instate municipality); 7) regional distribution (central west; south/southeast; or north/northeast); 8) self-declared skin color (white or non-white); 9) sexual maturity (prepubescent, pubescent, or postpubescent; 10) nutritional status; Ideal range, overweight, or obesity.

### Measurements

#### Stages of sexual maturation

The stage of sexual maturity was self-reported by the adolescents with the use of figures based on the criteria proposed by Tanner [15]. The stage of sexual maturation was self-reported by the adolescent himself, using figures indicative of the criteria proposed by Tanner [15]. For females, sexual maturity was assessed according to the growth of pubic and breast hair, for males it was assessed according to the growth of pubic and genital hair. Soon after, it was categorized into three categories of sexual maturation: Stage (I) = Pre-pubertal, Stages (II, III and IV) = Puberty and Stage (V) = Post-pubertal [15].

#### Anthropometric and nutritional status

Nutritional status was categorized using the body mass index [BMI = body mass (kg)/ height (m)^2^] in z-scores using the cutoff points described by De Onis et al. [16] < -2 (underweight); ≥ -2 and ≤ 1 (ideal range); > 1 and ≤ 2 (overweight); > 2 (obesity). Body mass was determined using an electronic scale (*Líder*) with a capacity of 200 kg and precision of 50 g. Height was determined using a portable stadiometer (*Alturexata*) with a capacity of 213 centimeters and precision of 1 mm. The tape was fixed to the wall and the individuals were placed in an upright position, with the upper limbs hanging along the body, and the heels, back and head leaning against the wall. For weight and height measurements, the adolescents were barefoot, wearing light clothing and in an orthostatic position [16].

#### Dependent variable

The insufficient practice of physical activity (dependent variable) was determined using the adapted Self-Administered Physical Activity Checklist, which has 24 types of physical activity.

The measure of physical activity (PA) was determined from the answers to two questions: mark the PA you practiced in the past week. You must include activities carried out at school as well as activities carried out outside of school [8]. For each of the physical activities you listed, you will be asked how many days a week and how much time a day, on average, you practiced in the past week. Twenty-four types of physical activities were analyzed and the time practiced per week for each leisure physical activity was quantified, consisting of days, hours and minutes. Then, the time and frequency in each leisure physical activity were multiplied and the sum of the times obtained was calculated [8].

The questionnaire has acceptable reproducibility (intraclass correlation coefficient = 0.88; 95% confidence interval: 0.84–0.91, with 52% agreement on meeting the recommended physical activity level and validity (Spearman’s ρ for total minutes per week of moderate and vigorous physical activitywas 0.62 against four 24-h recalls) in Brazilian adolescents [8].

For the present study, only those related to leisure activities (21 items) were analyzed. The time and frequency of each physical activity were quantified (days, hours, and minutes) and the sum of the total time spent on these activities was calculated [8].

Adolescents who spent more than 300 minutes per week on these activities were classified as sufficiently active at leisure and those who spent less than 300 minutes per week were classified as insufficiently active at leisure [8, 17]. The questionnaire was validated in the version of the ERICA study for the population of Brazilian adolescents, as suggested by Farias Júnior [18].

#### Ethical aspects

The ERICA study received approval from the institutional review board of each of the 27 states of Brazil. Erica was approved by the Ethics Committee of the Federal University of Rio de Janeiro, as well as by each state and the Brazilian Federal District, totaling 27 Research Ethics Committee.

In addition, before the start of data collection in the participating institutions, the signature of the Director’s Authorization Term for inclusion of students belonging to the school was requested. Parents and adolescents were informed about the study, and only adolescents who signed the Informed Consent Form, and parents or guardians who agreed to participate and signed the Informed Consent Form were included in the study. The adolescents signed a statement of consent and the parents/guardians signed a statement of informed consent.

#### Statistical analysis

Statistical analyses were performed with the aid of the STATA program (version 14.0) and the use of the *“Survey”* module due to the complex sampling design of the ERICA study. Descriptive statistics (absolute and relative frequencies) were employed. Bivariate analysis was performed with Pearson’s chi-square test and the linear trend chi-square test to determine the prevalence of insufficiently active adolescents according to the independent variables.

**Fig 1:**
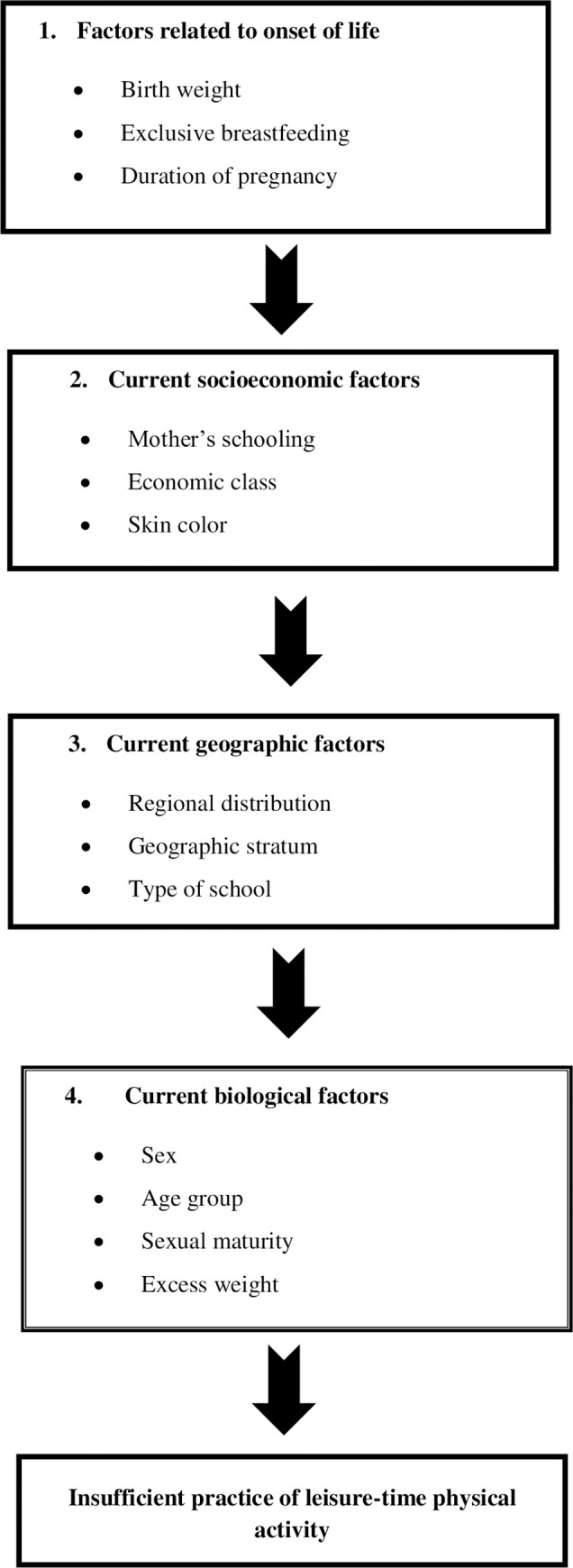
Theoretical model of determination of insufficient practice of leisure-time physical activity among Brazilian adolescents. Factors related to the onset of life (most distal level of the model) exerted an influence on the second and third blocks of variables, which were current sociodemographic factors (more proximal levels) that exerted a direct influence on the insufficient practice of physical activity by the adolescents.

Poisson regression analysis with robust variance was performed involving variables with p-value < 0.20 in the bivariate analysis for each outcome. Variables on the first hierarchical level were analyzed and those on the subsequent levels were incorporated into the model successively without subtracting the previously analyzed variables, using the theoretical model presented in Fig 1. Factors related to the onset of life (most distal level of the model) exerted an influence on the second and third blocks of variables, which were current sociodemographic factors (more proximal levels) that exerted a direct influence on the insufficient practice of physical activity by the adolescents (Fig 1). At the end of the analysis, only variables with a p-value <0.05 were considered significantly associated with the outcome. The results were expressed as prevalence ratios (PR) and respective 95% confidence intervals (CI).

## Results

Among the total sample of 36,956 adolescents, which was representative for an estimated 6,628,961 individuals of the target population, most were female (50.2%), between 15 and 17 years of age (53.4%), had self-declared non-white skin color (58.1%), and were in the pubescent stage (62.4%). Regarding regional characteristics and type of school, there was a greater proportion of students distributed in the south/southeast region (65.0%), residents of instate municipalities (58.1%), and students enrolled in the public school system (77.7%) (Table 1).

**Table 1:** Sociodemographic characteristics, type of school, and excess weight among Brazilian adolescents, ERICA STUDY 2013–2014.

Variables	Sample	Estimate of adolescents	%	95% CI
**Regional distribution**				
Central West	5,408	525,340	8.0	7.7–8.0
North/Northeast	18,478	1,794,092	27.0	26.9–27.2
South/Southeast	13,070	4,309,529	65.0	64.8–65.1
**Geographic stratum**				
Metropolis	27,302	2,777,952	41.9	41.7–42.1
Instate municipality	9,654	3,851,009	58.1	57.9–58.2
**Type of school**				
Public	27,223	5,150,058	77.7	72.3–82.2
Private	9,733	1,478,902	22.3	17.7–27.6
**Sex**				
Male	14,786	3,304,088	49.8	49.7–49.9
Female	22,170	3,324,873	50.2	50.0–50.3
**Age group (years)**				
12–14	16,921	3,089,012	46.6	46.4–46.7
15–17	20,035	3,539,949	53.4	53.2–53.5
**Sexual maturity**				
Prepubescent	168	30,985	0.5	0.03–0.05
Pubescent	22,873	4,137,158	62.4	61.0–63.7
Postpubescent	13,894	2,457,947	37.0	35.7–38.4
No information	21	2,869	0.1	0.02–0.08
**Skin color**				
Nonwhite	22,898	3,849,930	58.1	56.2–59.9
White	13,230	2,636,788	39.8	37.8–41.7
No information	828	142,241	2.1	1.8–2.5
**Mother’s age during pregnancy (years)**				
< 25	12,600	2,185,774	33.0	31.1–34.8
25 to 35	12,057	2,349,024	35.4	33.2–37.6
> 35	2,720	570,253	8.6	7.7–9.5
No information	9,579	1,523,908	23.0	19.5–26.7
**Mother’s schooling (years)**				
< 4	2,568	617,810	9.3	7.7–11.1
4 to 8	6,404	1,309,764	19.8	17.9–21.6
>8	17,470	2,968,979	44.8	41.5–48.0
No information	10,514	1,732,406	26.1	22.9–29.6
**Socioeconomic class**				
High	3,414	513,897	7.8	6.6–8.9
Middle	19,618	3,511,494	53.0	51.5–54.4
Low	2,790	450,018	6.8	6.1–7.5
No information	11,134	2,153,550	32.4	31.0–33.9
**Nutritional status**				
Ideal range	27,410	4,852,340	73.2	71.6–74.6
Overweight	6,515	1,165,442	17.6	16.4–18.7
Obesity	3,031	611,177,983	9.2	8.4–10.0
**Leisure-time physical activity**				
Sufficiently active	15,767	2,993,467	45.2	44.0–46.2
Insufficiently active	21,189	3,635,493	54.8	53.7–55.9

**Note:** CI: confidence interval; Socioeconomic class: High = subcategories A1 and A2; Middle = subcategories B1, B2, and C1; Low = subcategories C2, D, and E. BMI/age: body mass index for age, classified as underweight (z-score <-2), ideal range (z-score ≥-2 and ≤+1), overweight (z-score >+1 and ≤ +2), obesity (z-score >+2), an*d severe obesity (z-score >+3)*.

There was a predominance of adolescents with a middle and low socioeconomic status (59.8%), whose mothers were between 25 and 35 years of age during pregnancy (35.4%), and whose mothers had > eight years of schooling (44.8%). Regarding biological and behavioral factors at birth, 43.0% of the adolescents were born with adequate birth weight, 42.5% received exclusive breastfeeding for three to six months, and 67.8% were born after nine to 10 months of gestation. Regarding current nutritional status, 73.2% were in the ideal range and the majority was classified as insufficiently active at leisure (54.8%) (95% CI: 53.7–55.9) (Table 1).

**Fig 2:**
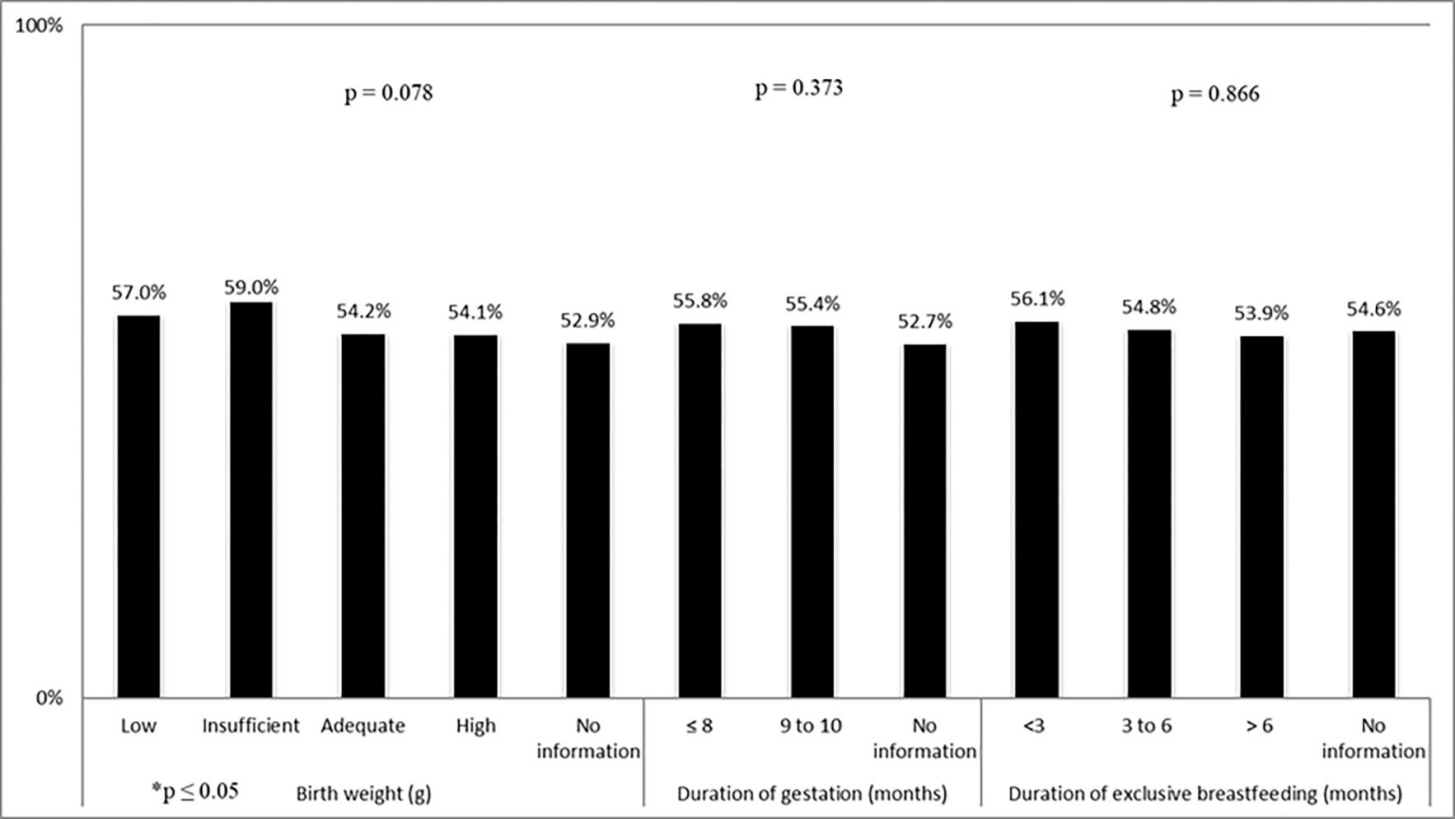
Prevalence of insufficient practice of leisure-time physical activity according to biological and behavioral factors at onset of life of Brazilian adolescents, ERICA STUDY 2013–2014. Birthweight: low (<2500g); insufficient (2500 to 2999g); adequate (3000g to 3999g), and high (≥ 4000g). Duration of pregnancy: <8 months and 9 to 10 months. Duration of exclusive breastfeeding: < 3 months, 3 to 6 months, and months.

Table 2 displays the prevalence of insufficient leisure-time physical activity according to sociodemographic characteristics and nutritional status. The prevalence of the outcome was significantly higher among individuals distributed in the southern/southeastern regions, the female sex, the higher age group, the low socioeconomic class, and the ideal range of nutritional status. In contrast, no significant associations were found between biological factors at the onset of life and the insufficient practice of leisure-time physical activity in adolescence, as illustrated in Fig 2.

**Table 2:** Prevalence of insufficient practice of leisure-time physical activity among Brazilian adolescents according to birth-related and current biological and sociodemographic factors, ERICA STUDY 2013–2014.

Variables	Insufficient practice of leisure-time physical activity	
	% (95% CI)	p
**Regional distribution**		<0.001
Central West	49.9 (46.7–53.1)	
North/Northeast	53.2 (51.6–54.8)	
South/Southeast	56.0 (54.5–57.5)	
**Geographic stratum**		0.9919
Metropolis	54.8 (53.7–55.9)	
Instate municipality	54.8 (51.1–58.5)	
**Type of school**		0.3193
Public	55.1 (53.9–56.4)	
Private	53.6 (50.8–56.3)	
**Sex**		<0.001
Male	39.1 (37.2–41.0)	
Female	70.4 (68.8–71.9)	
**Age group (years)**		<0.001
12–14	51.4 (49.8–53.0)	
15–17	57.8 (56.3–59.2)	
**Sexual maturity**		0.0369
Prepubescent	52.9 (40.9–64.6)	
Pubescent	53.7 (52.1–55.3)	
Postpubescent	56.7 (55.0–58.4)	
No information	43.9 (16.1–76.2)	
**Skin color**		0.9786
Nonwhite	54.9 (52.9–56.8)	
White	54.8 (52.8–56.7)	
No information	54.0 (46.2–61.7)	
**Mother’s age during pregnancy (years)**		0.4922
< 25	54.7 (52.3–57.1)	
25 to 35	56.1 (53.4–58.8)	
> 35	52.8 (48.5–57.0)	
No information	53.6 (50.9–56.3)	
**Mother’s schooling (years)**		0.2254
< 4	59.5 (53.6–65.1)	
4 to 8	55.7 (53.3–58.0)	
>8	54.0 (51.9–56.1)	
No information	53.8 (51.0–56.6)	
**Socioeconomic class**		<0.001
High	42.3 (38.6–46.1)	
Middle	54.5 (52.8–56.1)	
Low	64.8 (60.7–68.7)	
No information	56.2 (54.4–58.0)	
**Nutritional status**		<0.001
Ideal range	55.9 (54.6–57.1)	
Overweight	52.9 (50.5–55.3)	
Obesity	50.0 (46.9–53.1)	
**Birth weight (g)**		0.0780
Low	57.0 (51.9–61.9)	
Insufficient	59.0 (55.6–62.4)	
Adequate	54.2 (52.4–56.1)	
High	54.1 (50.5–57.7)	
No information	52.9 (50.1–55.7)	
**Duration of pregnancy (months)**		0.3730
≤8	55.8 (49.6–61.8)	
9 to 10	55.4 (53.7–57.2)	
No information	52.7 (49.9–55.5)	
**Exclusive breastfeeding (months)**		0.8663
<3	56.1 (53.0–59.1)	
3 to 6	54.8 (52.8–56.7)	
>6	53.9 (49.0–58.8)	
No information	54.6 (52.4–56.7)	

**Note:** CI: confidence interval; Socioeconomic class: High = subcategories A1 and A2; Middle = subcategories B1, B2, and C1; Lo*w = subcategories C2*, *D*, *and E*. BMI/age: body mass index for age, classified as underweight (z-score <-2), ideal range (z-score ≥-2 and ≤+1), overweight (z-score >+1 and ≤ +2), obesity (z-score >+2), an*d severe obesity (z-score >+3)*. Birthweight: low (<2500g); insufficient (2500 to 2999g); adequate (3000g to 3999g), and high (≥ 4000g). Duration of pregnancy: <8 months and 9 to 10 months. Duration of exclusive breastfeeding: < 3 months, 3 to 6 months, and months.

Table 3 displays the results of the simple and multiple Poisson regression analyses testing associations between the insufficient practice of leisure-time physical activity and biological/sociodemographic variables at birth and current factors. Adolescents in the low and middle classes had a 35% and 21% greater likelihood of being insufficiently active, respectively. Regarding the regional distribution, adolescents residing in the southern/southeastern regions were 11% more likely to be insufficiently active at leisure compared to those who resided in the central western region. Older adolescents (15–17 years) were 10% more likely to be insufficiently active compared to younger adolescents (p<0.001) and girls were 77% more likely to be insufficiently active at leisure than boys (p<0.001). Moreover, adolescents diagnosed with overweight and obesity were less likely to be insufficiently active at leisure (Table 3).

**Table 3:** Associations between birth-related/current biological/sociodemographic variables and insufficient practice of leisure-time physical activity among Brazilian adolescents, ERICA STUDY 2013–2014.

Variables	Insufficient practice of leisure-time physical activity		
	Crude analysis	Adjusted analysis	
	PR (95% CI)	PR (95% CI)	p
**Level 1 –Birth weight (g)**			
Adequate	1	1	
Low	1.05 (0.96–1.14)	1.01 (0.92–1.11)	0.719
Insufficient	1.08 (1.01–1.16)	1.03 (0.97–1.09)	0.282
High	0.99 (0.92–1.07)	1.01 (0.94–1.08)	0.671
No information	0.97 (0.90–1.04)	0.99 (0.92–1.06)	0.822
**Level 2 –Socioeconomic class**			
High	1	1	
Low	1.53 (1.37–1.69)	1.35 (1.21–1.50)	<0.001
Middle	1.28 (1.16–1.42)	1.21 (1.10–1.33)	<0.001
No information	1.32 (1.21–1.45)	1.27 (1.16–1.39)	<0.001
**Level 3 –Regional distribution**			
Central West	1	1	
North/Northeast	1.06 (0.99–1.14)	1.06 (0.98–1.14)	0.092
South/Southeast	1.12 (1.04–1.20)	1.11 (1.03–1.19)	0.002
**Level 4 –Sex**			
Male	1	1	
Female	1.79 (1.69–1.90)	1.77 (1.68–1.88)	<0.001
**Level 4 –Age group (years)**			
12–14	1	1	
15–17	1.12 (1.07–1.16)	1.10 (1.06–1.14)	<0.001
**Level 4 –Sexual maturity**			
Postpubescent	1	1	
Prepubescent	0.93 (0.74–1.17)	1.03 (0.84–1.26)	0.758
Pubescent	0.94 (0.90–0.99)	0.97 (0.93–1.01)	0.237
No information	0.77 (0.35–1.70)	0.97 (0.47–1.98)	0.937
**Level 4 –Nutritional status**			
Ideal range	1	1	
Overweight	0.94 (0.90–0.99)	0.95 (0.91–1.00)	0.057
Obesity	0.89 (0.83–0.95)	0.93 (0.87–1.00)	0.046

*Level 2 adjusted by Level 1

*Level 3 adjusted by Levels 1 and 2

*Level 4 adjusted by Levels 1, 2 and 3.

Note: PR = prevalence ratio; CI = confidence interval.

Birthweight: low (<2500g); insufficient (2500 to 2999g); adequate (3000g to 3999g), and high (≥ 4000g). Socioeconomic class: High = subcategories A1 and A2; Middle = subcategories B1, B2, and C1; Low = subcategories C2, D, and E. BMI/age: body mass index for age, classified as underweight (z-score <-2), ideal range (z-score ≥-2 and ≤+1), overweight (z-score >+1 and ≤ +2), obesity (z-score >+2), an*d severe obesity (z-score >+3)*.

## Discussion

As the main findings of the present study, the factors associated with the insufficient practice of leisure-time physical activity in adolescence were the low and middle economic classes, residing in the southern/southeastern regions of the country, the female sex, and the older age group (15 to 17 years). Moreover, adolescents with excess body weight (obesity) were less likely to be insufficiently active at leisure.

The results of the ERICA study revealed that more than half of Brazilian adolescents are insufficiently active. Systematic reviews [19, 20] of studies involving Brazilian adolescents report that the prevalence of physical inactivity ranges from 25.1% to 93.0%. The majority of studies in these reviews used a questionnaire as the measurement tool. Thus, a possible explanation for this wide prevalence range may be the use of non-standardized methods and non-validated instruments as well as the investigation of physical activity in different domains (manner of arriving at school, leisure-time physical activity, etc.), which hinders comparisons of the findings. Data from the National Student Health Survey conducted in 2015 based on data collected from questionnaires indicated that 60.8% of adolescents were classified as insufficiently active and 4.8% were classified as inactive, with higher proportions found more often in the female sex and students enrolled at public schools [7].

One of the aims of the present study was to determine which factors at the onset of life may be associated with the insufficient practice of physical activity in adolescence. Although a recent study found that the maintenance of the practice of physical activity in childhood and adolescence is associated with environmental and biological factors at the onset of life [9], this association does not yet seem to be clear in the literature. In a study conducted by Mattocks et al. [21], the physical activity level of children between 11 and 12 years of age was influenced little by factors to which these children were exposed at early ages, which is in agreement with the results of the present investigation. The international literature offers conflicting results regarding the association between a low birth weight and physical activity level in adolescents [9, 22]. While Hallal et al. [5] and Pearce et al. [23] found no significant association, Tikanmäki et al. [24] found that children born with a high birth weight and whose parents/mothers were obese had a greater likelihood of being insufficiently active and having low physical fitness.

In the present study, factors related to the onset of life seem not to be associated with the behavior of adolescents with regards to the insufficient practice of physical activity, which is a noteworthy result, as physical inactivity is a risk factor for chronic non-communicable diseases that could be acquired throughout life [1] However, little is known on this subject due to the lack of studies involving this subgroup of the population as well as methodological and logistic difficulties in assessing these late effects of exposure to early factors on low physical activity levels in this phase of life. Moreover, the results can be influenced of current factors/determinants of the environment to which adolescents are exposed [5]. Thus, longitudinal studies are needed to evaluate the possible effects of birth-related variables on physical activity and possible interrelations in adolescence [24].

Other factors associated with the insufficient practice of leisure-time physical activity in the present study are consistent with data reported in the scientific literature [7]. It is well documented in the literature that girls are less physically active than boys, especially with regards to vigorous activities [6, 7, 19, 20]. This divergence may reflect a mode of life based on a patriarchal society, in which boys were more encouraged and received more social support for engaging in physical activities, whereas girls were responsible for domestic activities, which could lead to an increase in sedentary behavior [25].

The insufficient practice of leisure-time physical activity was associated with the low and middle socioeconomic classes of the adolescents in the present study. Ceschini et al. [26] report similar results in a study involving 1899 male and female adolescents (15 to 20 years of age) in the city of São Paulo. The authors found a high frequency (63.9%) of physical inactivity (<300 minutes per week) among students who studied in the evening and had lower socioeconomic levels (Classes D and E). A plausible explanation would be related to the lower buying power of the families of these adolescents, which may interfere with the acquisition of knowledge regarding a healthy lifestyle as well as limit access to the practice of leisure-time physical activity due to the lack of infrastructure in cities and neighborhoods (presence of public squares, parks, bicycle lanes, etc.) [27]. Another hypothesis regards the fact that low-income adolescents enter the job market at a younger age in an attempt to improve the socioeconomic situation of the family and this tends to diminish the amount of free time, which can lead to greater physical inactivity [6, 8].

It is well documented in the literature that the insufficient practice of leisure-time physical activity is influenced by current factors/determinants in adolescence. Geographic distribution is one such factor, as the prevalence of this outcome is higher in states located in the northern/northeastern and southern/southeastern regions [8]. This higher prevalence may be analyzed based on regional and socioeconomic inequalities in the particularities of each sub-region as well as the type of measure used, the cutoff points adopted, and different associated factors and/or determinants of the insufficient practice of leisure-time physical activity [28].

An association was found between age and the insufficient practice of leisure-time physical activity in the present investigation. This finding is compatible with data from a large part of epidemiological studies conducted with adolescents, which indicate that the prevalence of insufficient practice of physical activity is higher among older adolescents [6, 8].

Interestingly, excess weight (obesity) was a protection factor against the insufficient practice of leisure-time physical activity among the adolescents analyzed. This finding is inconsistent with data from a previous study, which reported an inverse association between the practice of physical activity and excess weight in adolescents [29]. Another study found no association between these variables [30]. A possible hypothesis for this result would be the fact that the questionnaire employed in the present study does not have sufficient discriminatory capacity to distinguish the practice of physical activity between adolescents in the ideal weight range and those with excess weight [18].

This study has limitations that should be considered. The cross-sectional design does not enable establishing cause-and-effect relationships. The non-response rate regarding biological and behavioral factors related to the onset of life was high (range: 30 to 40%), which may be attributed to possible recall bias. In an attempt to minimize this limitation, “no information” categories were created for all missing variables. Another limitation was the measurement of the duration of pregnancy in months rather than weeks.

The present findings are worrisome, as adolescence is an important period for the regular practice of physical activity and unhealthy habits in this phase of life can be perpetuated in subsequent phases [6, 7, 29]. Thus, there is a need to implement strategies focused on physical activity at schools, especially physical education classes, as well as social support from family and friends to stimulate the practice of physical activity among adolescents [25].

In summary, when taking into account the possible associations found between the factors at the beginning of life with food consumption and physical inactivity, it is clear that this work was important considering that it has been demonstrated in the literature that a possible mechanism of association between the low birth weight and the increased risk of developing NCDs, such as obesity and cardiovascular diseases, can be explained by changes in energy balance related to health behaviors, including increased food consumption, inactivity physical activity and sedentary behavior [3]. When considering these correlates, it is worth emphasizing the importance of support from basic education teachers, This analysis is necessary for the improvement of maternal and child care programs and/or assistance, with regard to the regular practice of physical activity, for example, a greater offer of different types of physical activity, especially in the school environment and during leisure time, which can be an attenuator of the adverse conditions of early life, and favor the reduction of predisposition to chronic diseases, such as obesity, in adolescence. It also contributes to the field of adolescent health by offering data for the development, implementation, and strengthening of prevention strategies and measures for combating metabolic disorders resulting from physical inactivity.

## Conclusion

It is concluded that the factors sociodemographic current associated with physical inactivity were the low and middle socioeconomic class, being distributed in the North/Northeast and South/Southeast, female and older (15–17 years). It was also observed that overweight and obesity were protective factors for physical inactivity. In contrast, biological and behavioral factors in early life were not significantly associated with physical inactivity in Brazilian adolescents.
